# Continuous ratings of movie watching reveal idiosyncratic dynamics of aesthetic enjoyment

**DOI:** 10.1371/journal.pone.0223896

**Published:** 2019-10-25

**Authors:** Ayse Ilkay Isik, Edward A. Vessel

**Affiliations:** Neuroscience Department, Max Planck Institute for Empirical Aesthetics, Frankfurt, Germany; Universidad de Tarapaca, CHILE

## Abstract

Visual aesthetic experiences unfold over time, yet most of our understanding of such experiences comes from experiments using static visual stimuli and measuring static responses. Here, we investigated the temporal dynamics of subjective aesthetic experience using temporally extended stimuli (movie clips) in combination with continuous behavioral ratings. Two groups of participants, a *rate* group (n = 25) and a *view* group (n = 25), watched 30-second video clips of landscapes and dance performances in test and retest blocks. The *rate* group reported continuous ratings while watching the videos, with an overall aesthetic judgment at the end of each video, in both test and retest blocks. The *view* group, however, passively watched the videos in the test block, reporting only an overall aesthetic judgment at the end of each clip. In the retest block, the *view* group reported both continuous and overall judgments. When comparing the two groups, we found that the task of making continuous ratings did not influence overall ratings or agreement across participants. In addition, the degree of temporal variation in continuous ratings over time differed substantially by observer (from slower “integrators” to “fast responders”), but less so by video. Reliability of continuous ratings across repeated exposures was in general high, but also showed notable variance across participants. Together, these results show that temporally extended stimuli produce aesthetic experiences that are not the same from person to person, and that continuous behavioral ratings provide a reliable window into the temporal dynamics of such aesthetic experiences while not materially altering the experiences themselves.

## Introduction

Aesthetically pleasing experiences, such as looking at a painting, listening to a piece of music, or watching a movie or dance performance, develop dynamically in time. These experiences involve complex processes generated by the interactions between perception, attention, decision making, affect and emotion–and, of course, an individual observer’s background knowledge [1,2]. However, little is known regarding the temporal processes giving rise to these experiences such as how much time is necessary for an aesthetic experience to develop or how these component processes interact in time to produce an aesthetic experience.

There is evidence suggesting that people are able to form stable aesthetic judgments on the basis of very brief exposures: ratings for 500 ms musical excerpts [3] and for 50 ms presentations of scenes [4,5] are highly correlated to ratings for more extended presentations. However, other studies suggest that understanding and appreciation of the objects or events that give rise to aesthetic experiences typically requires more time. For example, average looking times for artworks in museums are reported to be around 20 seconds, with large variations across people and artworks [6–8]. It also takes time for an aesthetic judgment to develop; for example, making a judgment on whether something is beautiful takes longer than making a perceptual judgment on whether something is symmetric [9].

Despite evidence for the temporally extended nature of aesthetic experiences, our current understanding derives mainly from experiments employing static materials and single, *post-hoc* summary judgments. In a typical experiment, participants are asked to make binary judgments such as whether they find an artwork beautiful or not or are asked to state the degree of liking or beauty on a Likert scale. Not only may such post-viewing summary judgments reflect a distorted memory of an experience [10–12], the underlying experience with even a static image may in fact be dynamic, and thus not well captured by a single value. For example, a person looking at an image or a scene may first focus on larger features before zooming in on specific details. Such shifts in attention to different regions or scales of an image are likely accompanied by shifts in felt emotion and aesthetic appraisal as new details are appreciated and integrated into a coherent understanding of the work. Two recent studies have explored such dynamics during the viewing of static visual images using continuous ratings of pleasure responses [13,14]. These studies observed an asymptotic rise to a peak level of pleasure starting with the image presentation followed by a steady-state plateau and then a falloff with a slow time constant after the image offset. These dynamics suggest that participants continued to report having pleasure even after the image was no longer on the screen.

At least two considerations motivate the use of dynamically changing stimuli, in addition to continuous behavioral measures, in the study of aesthetic experiences. Practically, many art forms are inherently dynamic (e.g. dance, film, music). More importantly, a theoretical characterization of the underlying nature of relevant mental processes (e.g. as linear or nonlinear in a dynamical systems sense) requires variability in the input. Here, we used temporally extended visual stimuli in combination with continuous behavioral ratings to characterize the temporal dynamics of subjective aesthetic experiences. Participants viewed 30-second video clips of two different categories (dance performances or landscapes) while continuously evaluating how much they *enjoyed* the clip at the present moment. In addition to the continuous evaluation, participants were also asked to make an overall aesthetic judgment at the end of each video clip. The use of dance and landscape stimuli, categories that are widely used in empirical aesthetics studies [15–19], additionally allowed us to test for generalization across visual experiences with quite divergent properties (e.g. bodies making intentional movements versus non-embodied environmental movements within intentionally framed landscape shots).

This design allowed us to address several outstanding theoretical issues concerning the collection and use of continuous data. The first consideration was whether it was possible to detect dynamic changes at all. A primary aim of this study was to inspect temporal changes in continuous response data and to characterize the degree and source of observed variations.

Furthermore, little is known about the impact of repeated presentation on continuous behavioral ratings. Some hypotheses, such as the mere exposure effect [20] or perceptual fluency [21,22], suggest that repeated exposure to a stimulus should result in increased liking. In contrast, other accounts suggest that repetition causes habituation or boredom, resulting in decreased liking [23,24]. A third possibility is that repeated exposure may have different effects for different stimulus types [25] or may lead to changing time courses as attention shifts to different aspects of a stimulus. We therefore assessed the test-retest reliability of continuous rating time series across two exposures.

An additional concern is that the very act of making a continuous judgment may affect the experience itself. As existing evidence for such interference is inconclusive [26,27], we included a direct test in our experimental design. Two separate groups participated in both test and retest sessions; while one (*rate*) group made continuous ratings in both sessions, the other (*view*) group made continuous ratings only in the retest session, but not the initial test session. If continuous judgments affect the experience, we would expect the overall judgments to differ across *rate* and *view* groups.

Finally, it is known that aesthetic judgments of visual artworks are highly idiosyncratic [17,28]. We investigated the degree to which dynamic visual stimuli lead to more similar aesthetic experiences in different individuals. Rather than averaging traces across participants and using averaged time courses for further analyses [29,30], we quantified the similarity of continuous rating traces across different participants and compared the measured agreement to that observed for overall summary judgments.

## Materials and methods

### Participants

Fifty participants took part in the experiment; twenty-five in the *rate* group (13 female; mean age = 27.92, STD = 8.74) and twenty-five in the *view* group (13 female, mean age = 28.16, STD = 7.75). All participants had normal or corrected-to-normal vision, gave informed consent as approved by the Ethics Council of the Max-Planck Society and were paid for their participation.

### Stimuli

Stimuli were 30 second artistic video clips of *landscapes* (n = 15) and *dance performances* (n = 15) that were collected from video streaming websites. The landscape videos were slow-motion, aerial drone shots or time-lapse photography depicting different types of natural landscapes (e.g. mountain, forest, ocean, river). The dance clips consisted of modern dance and ballet performances. To ensure that aesthetic engagement was mainly driven by content belonging to the chosen category, we picked dance clips with plain backgrounds and landscape clips without human beings or other objects.

Video clips were chosen from a larger pool of videos (*landscapes*, n = 26, *dance performances*, n = 26) that were pretested in a pilot study in which 21 participants watched and rated the videos similar to the current study. Dance and landscape videos for the current experiment were selected by matching the degree of across-participant variance on a stimulus by stimulus basis for the overall aesthetic ratings of the two sets of videos. All video clips had the same aspect ratio (16:9), resolution (1280x720 px), and were saved using the same video compression method (H.264). Stimuli were presented using PsychoPy [1.84.2] [31] and MovieStim3 at a distance of 60 cm (approximate field-of-view 28^o^ horizontal by 18^o^ vertical). As the rights for many of the clips are privately held and thus we do not have permission to distribute them, the complete stimulus set cannot be made publicly available. However, one shorter but representative example video clip for each category can be found in supplementary materials (S1 Movie, S2 Movie).

### Procedure

**Fig 1:**
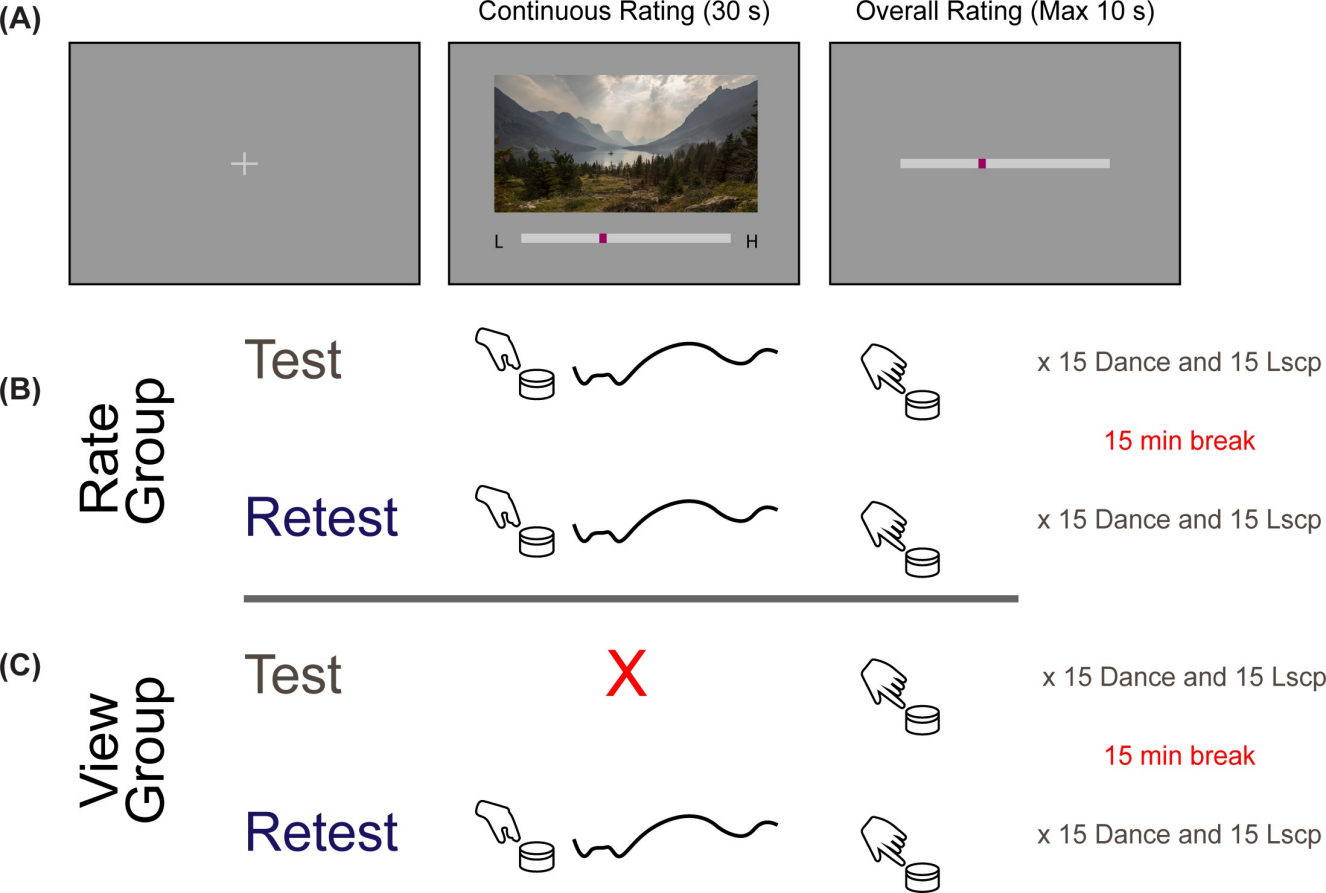
Schematic description of measuring continuous aesthetic responses to dynamically changing visual experiences. (A) Participants viewed videos of landscapes or dance performances for 30 seconds while making continuous ratings of the moment-to-moment enjoyment they were having. This was immediately followed by an overall rating indicating the intensity of their aesthetic experience of the clip. Both types of responses were made using a dial that controlled the slider display on the screen. (B) The *rate* group completed continuous and overall ratings in both test and retest sessions. (C) The *view* group gave overall ratings but not continuous ratings in the test session. In the retest session, participants in this group performed both types of ratings.

Participants sat in front of a computer screen in a sound isolated behavioral chamber and viewed the videos in complete darkness. In this study, we had two groups of participants. The first group of participants (*rate* group) carried out the same task across test and retest sessions: they made continuous evaluations while watching the video as well as giving an overall aesthetic rating at the end of the video (Fig 1B). However, the second group of participants (*view* group) watched the videos without making continuous evaluations in the test session, followed by an overall summary judgment at the end of the video clip. In the retest session, however, the *view* group performed both continuous and overall rating tasks (Fig 1C). In both versions, the stimuli were presented in blocks of landscape and dance videos. The order of the movies in the blocks were randomized across test and retest sessions. The order of dance and landscape blocks differed across participants but was the same for one participant across two sessions. Both sessions took place on the same day. After the first session, the participants were given a break of about 15 minutes during which they filled out several questionnaires including a background questionnaire, the Snaith-Hamilton Pleasure Scale (SHAPS) [32], the Positive and Negative Affect Schedule (PANAS) [33], the State Trait Anxiety Inventory (STAI) [34] and the Aesthetic Responsiveness and Engagement Assessment (AREA; Wallot et al., under review) a scale to measure trait-level aesthetic responsiveness. Participants used a dial to give ratings (PowerMate, Griffin Technology, New York). This dial controlled the position of a cursor moving horizontally on a scale placed under the video. At the beginning of the trial the cursor appeared in the middle of the scale indicating a neutral rating. The right and left ends of the scale were denoted with letters L and H corresponding to low and high aesthetic appeal (Fig 1A). Participants were instructed to give ratings of their subjective aesthetic experience of the clips, that there were no right or wrong answers, and that they should base their judgments on their personal experience not on other video clip characteristics (e.g. video quality). For the continuous evaluation task participants were instructed to move the cursor to the right if they were feeling more enjoyment or pleasure and to move the cursor to the left if they were feeling less enjoyment or pleasure when watching the video clip. The exact German instruction was: “Wie sehr Ihnen das Video in jedem Moment gefällt?” (“How much are you enjoying the clip at each moment?”). After the video clip finished playing, participants were shown a similar scale appearing in the middle of the screen and asked to make a summary aesthetic judgment, “Wie intensiv hat Sie das Video insgesamt angesprochen?” (“How intense was your aesthetic experience overall?”). Participants were told that they might have a more intense aesthetic experience for many different reasons, such as a clip being experienced as beautiful, profound or emotionally moving. This form of summary aesthetic judgment has proven effective in several previous studies as a way to measure the intensity of aesthetic experiences in a single judgment despite the potential variety of such experiences [17,35]. While it is possible that this form of aesthetic judgment may miss important subtleties between different types of aesthetic experiences, it captures the major axis of variation, including beauty [36] while also capturing some of the more complex forms of aesthetic experience that may not be “beautiful” in the strict sense (such as being moved, e.g. [37]. At the end of the second session participants also answered a small questionnaire and reported how much they like natural landscapes and dance performances, more generally (1 to 5 scale).

### Analysis

Using the timestamps of changes in participants’ ratings, time series were created with a sampling rate of 10 Hz (300 data points per video). Both overall and continuous ratings were mapped such that the maximum position on the slider was coded as 1 and minimum position was coded as -1.

#### Linear mixed-effects models

We ran linear mixed-effects models (LMMs) with the lmer function from *lme4* [38] implemented in R (version 3.4.3) to make several comparisons in our data. We chose the LMM approach because it allows between-participant and between-stimuli variance to be estimated simultaneously, yielding advantages over conventional multiple regression analysis. Most importantly, we choose to use LMMs because of the method’s superiority in estimating not only the fixed effect parameters but also the parameters of the variance and the covariance parameters of random effects due to participants [39]. Our experimental factors consisted of two levels and we defined sum contrasts to make critical comparisons across them. We used Principal Components Analysis (PCA) of the random-effects variance-covariance estimates for each fitted mixed-effects model to prevent overparameterization [40]. Random slopes not supported by the PCA and not contributing significantly to the goodness of fit (as shown by likelihood ratio tests) were removed from the model. We report regression coefficients along with a *t* statistic applying a two-tailed criterion (|*t*| ≥ 1.96), corresponding to a 5% error criterion for significance. To break down significant interactions, the lsmeans package [41] was used to obtain least-squares means and perform Tukey adjusted comparisons of factor levels whenever applicable.

#### LMM for overall ratings

Main effects of category, session, and group on the overall ratings were tested using linear mixed effects analysis. Category, session and group were entered as fixed effects. Intercepts for participants and stimulus items were included as random effects, as well as by-participant random slopes for the effect of category. With this model we sought to investigate three things: first, whether ratings differed by category (dance, landscape); second, whether the overall ratings were different across sessions (test, retest) and third, whether the two groups (*rate* vs *view*) differed in their ratings across sessions.

#### LMMs for MM1 agreement scores

We calculated two LMM analyses for agreement scores (see agreement analysis section below); one with the MM1 scores for overall ratings and one with the MM1 scores for continuous ratings. In the first LMM (MM1 for overall ratings), category, session and experiment group were entered as fixed effects and participant-wise intercepts and slopes were entered as random effects for category and session. In the second LMM (for continuous ratings), category, session and experiment groups were entered as fixed effects. Intercepts for participants and stimulus items were included as random effects, as well as participant-wise random slopes for the effect of category. In this LMM, the *view* group had MM1 scores only from the retest session as our design did not include continuous ratings in the test session.

#### LMMs for root mean squared differences (rmsd)

Temporal variability in continuous rating traces were quantified using a root mean squared difference measure (see Results, below). Following inspection of the distribution/residuals and the power coefficient output of the boxcox procedure [42], rmsd scores were log-transformed in order to more closely approximate a normal distribution and meet LMM assumptions. For this LMM, category and experiment group were included as fixed effects, random intercepts for participants and movies were included as random effects, as well as participant-wise random slopes for the effect of category and session. We did not include session as a factor because only retest session scores were included in this LMM (as a separate LMM for the *rate* group’*s* test and retest scores did not result in a significant session effect).

#### Agreement analysis

Agreement for overall and continuous ratings across participants was quantified by using a “mean-minus-one” (MM1) correlation measure [17]. To calculate the MM1 scores for overall ratings we took each individual’s ratings and computed Pearson correlations with the average ratings of all other (N-1) individuals. This was done separately for each factor level (dance|test, dance|retest, landscape|test, landscape|retest), producing four *r* scores for each individual that indicated how much this person agreed with the rest of the participants for this category and session. We applied a similar procedure to calculate the MM1 ratings for continuous ratings. This time, we correlated one participant’s rating time course for one movie with the averaged time course for the same movie across all other participants. That way, we obtained one agreement value per video clip and participant indicating how much in agreement this person was with the rest of the participants for their moment-to-moment ratings for that movie clip.

To obtain average across-observer MM1 scores, we first transformed individual *r*-values to *z*-values, computed the mean and 95% confidence intervals, and then transformed those scores back to *r*-values since this method has been shown to result in less biased estimates than averaging raw correlations [43,44].

As a comparison to MM1, we also employed a variance decomposition method [45] by first partitioning the total variance of responses into non-repeatable vs repeatable variance and then subdividing the repeatable variance into shared vs individual variance in responses.

## Results

**Fig 2:**
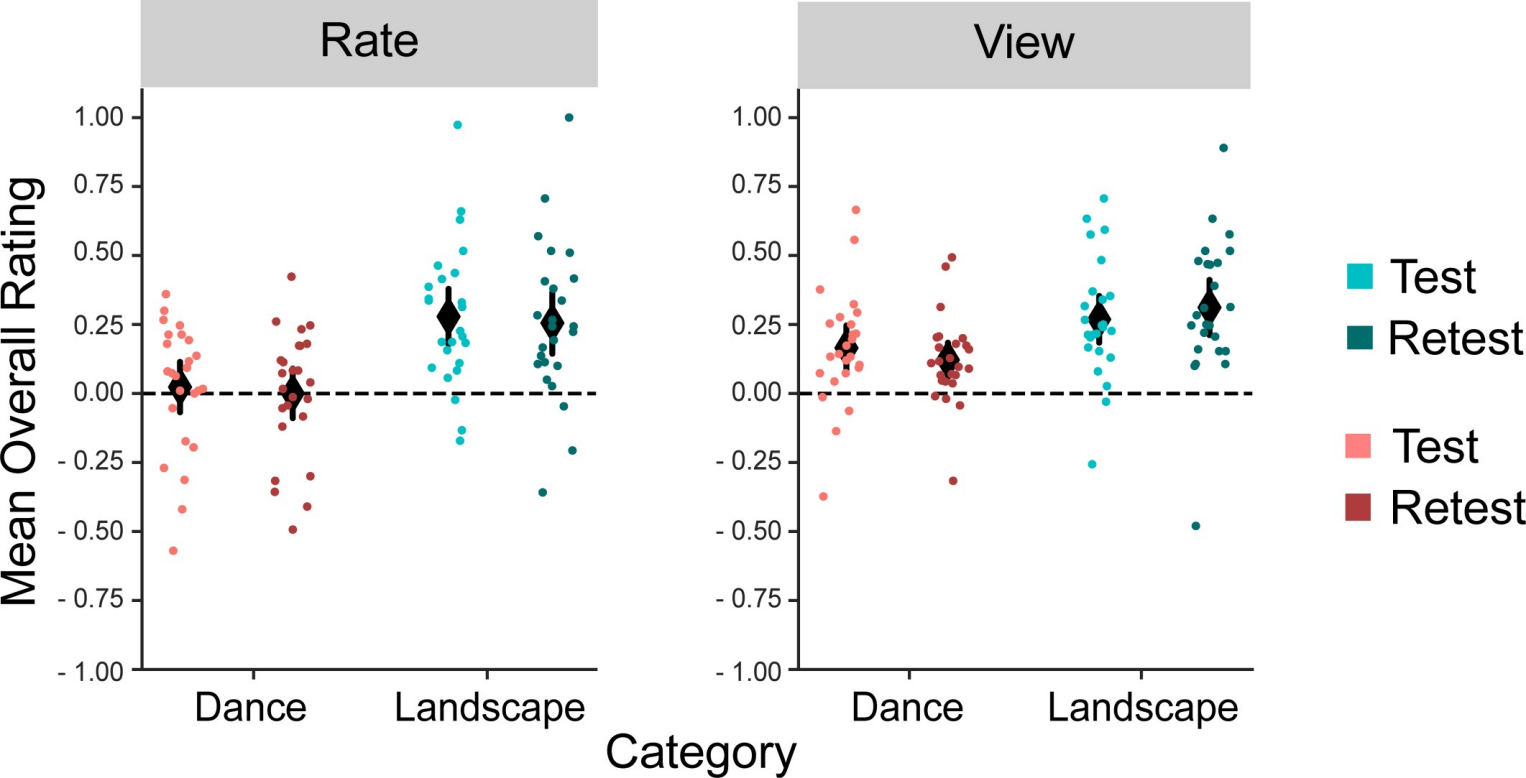
Distributions of mean overall ratings across groups (*rate* vs. *view*), sessions (test-retest) and categories (dance vs. landscape). On average, landscape videos were rated higher than dance videos, but there was no main effect of session or group (The diamond symbols show the means and black vertical lines indicate 95% confidence intervals of the means).

An LMM regression analysis of the overall ratings revealed a main effect of stimulus category (B = -0.10, *SE* = 0.04, *t* = -2.47, *p* = 0.017)indicating that overall ratings for landscape videos were higher compared to dance videos (Fig 2) (Table 1). However, there were no main effects of session (*t* = 0.79, *p* = 0.43) or *view/rate* group (*t* = -1.90, *p* = 0.064), and no interactions (Table 1). To the degree that overall ratings can serve as a proxy for the subjective nature of aesthetic experience, this suggests that making continuous ratings did not influence the overall quality of participants’ aesthetic experiences.

**Table 1:** Results of the linear mixed-model for overall ratings.

Fixed effects	*Estimate*	*SE*	*CI*	*t*	*p*
(Intercept)	0.18	0.04	0.10–0.25	4.61	**<0.001**
Session (Test vs Retest)	0.01	0.01	-0.01–0.02	0.79	0.430
Category (Dance vs Landscape)	-0.10	0.04	-0.18 –-0.02	-2.47	**0.017**
Group (Rate vs View)	-0.04	0.02	-0.08–0.00	-1.90	0.064
Session x Category	0.01	0.01	0.00–0.03	1.45	0.148
Session x Group	0.01	0.01	-0.01–0.02	0.77	0.444
Category x Group	-0.03	0.02	-0.07–0.02	-1.13	0.265
Session x Category x Group	-0.01	0.01	-0.03–0.00	-1.46	0.143
**Random Effects**	** **	** **	** **	** **	** **
Residual variance (σ^2^)	0.16	By participant variance in category (τ11)			0.03
Random intercept variance by participant	0.02	Random slope and intercept correlation (ρ01)			-0.26
Random intercept variance by item	0.03				
Marginal R^2^ / Conditional R^2^ *	0.050 / 0.351				

* Marginal: Variance explained by the fixed factors, Conditional: Variance explained by the fixed and random factors

To make sure that the two stimulus categories did not contain different degrees of average motion energy we derived a measure of motion energy for each video by applying a Gabor jet simple cell model ([46] to each frame and then computing a vector of framewise differences in the model output. This model was used as opposed to a simpler pixelwise motion energy metric because it more closely resembles the information thought to be encoded by the early visual system [47]. A Kolmogorov-Smirnov test was applied to the average motion energy values across dance and landscape categories. The test results supports the conclusion that these two samples are drawn from the same population (*ks statistic = 0*.*40*, *p* = 0.18).

**Fig 3:**
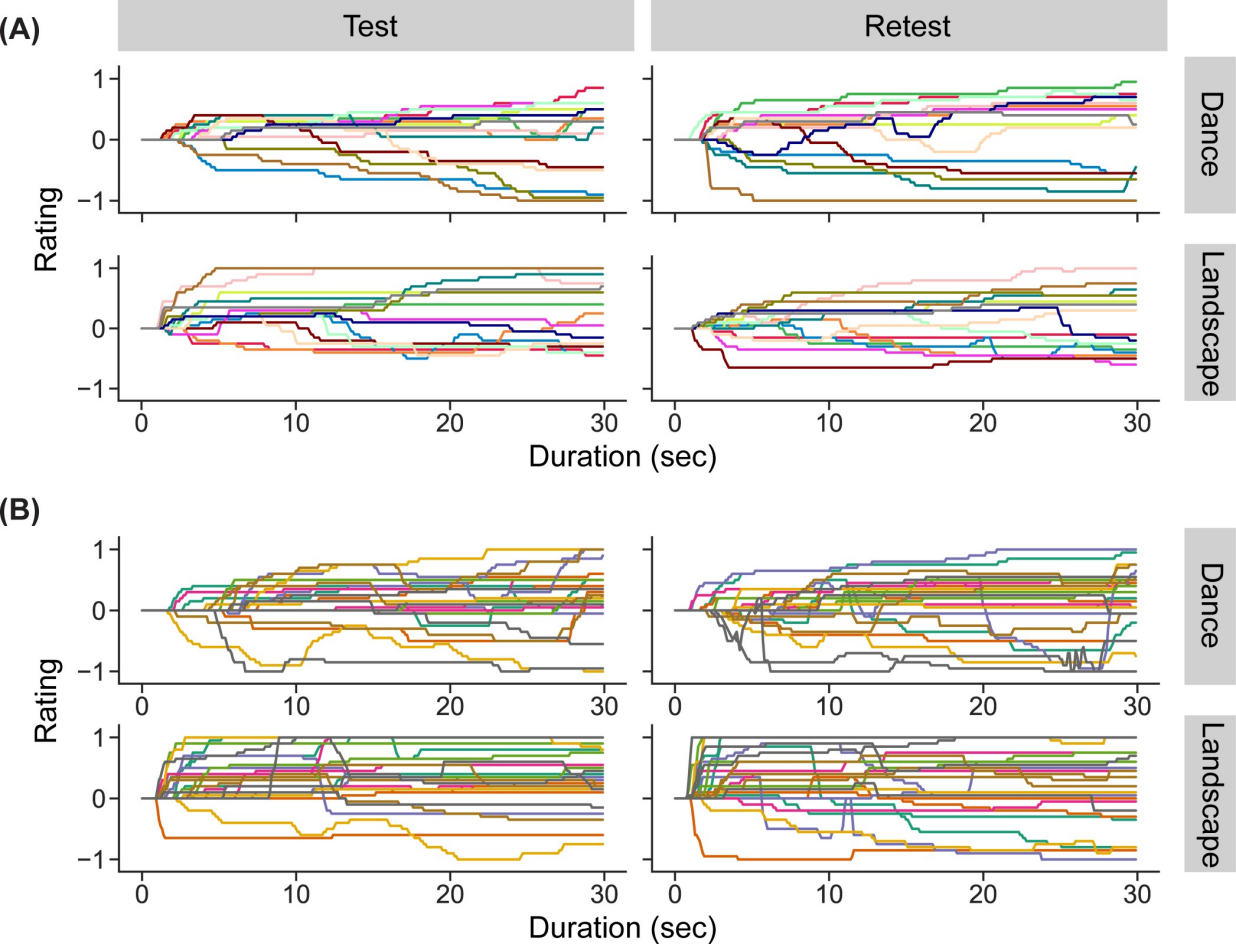
Representative continuous rating traces. (A) Continuous rating traces from one participant for each movie clip. In addition to differences in overall mean liking, some movies elicited more temporal dynamics than others. Rows show different categories (Dance, Landscape) and columns show ratings for different sessions. (B) Continuous rating traces given by each participant for one dance and one landscape clip. These movie clips were rated remarkably differently by different participants, both in terms of its overall mean liking, but also in terms of variability over time. Each row shows a sample movie from one genre (Dance, Landscape) and columns show ratings for different sessions.

Examination of trial-by-trial continuous rating traces revealed large differences in individual responses, both across movies but also across participants. Within each participant, some clips were rated more dynamically than others (Fig 3A). In addition, different participants generated widely divergent continuous rating profiles for the same movie, ranging from strongly liked to strongly disliked, and from mostly static to strongly dynamic (Fig 3B). Surprisingly, there were even disagreements about the valence of specific moments, with some observers increasing their reported enjoyment at the same moment other observers decreased their reported enjoyment.

We characterized this variability in three different ways. First, we measured the degree to which an individual observer responded to the same video in the same manner on repeated viewing (test-retest reliability). Second, we quantified the degree of temporal variability in individual continuous rating traces. Third, we measured the degree to which different observers responded similarly when watching the same video (across-observer agreement).

### Reliability of overall and continuous judgments upon repeated viewing

**Fig 4:**
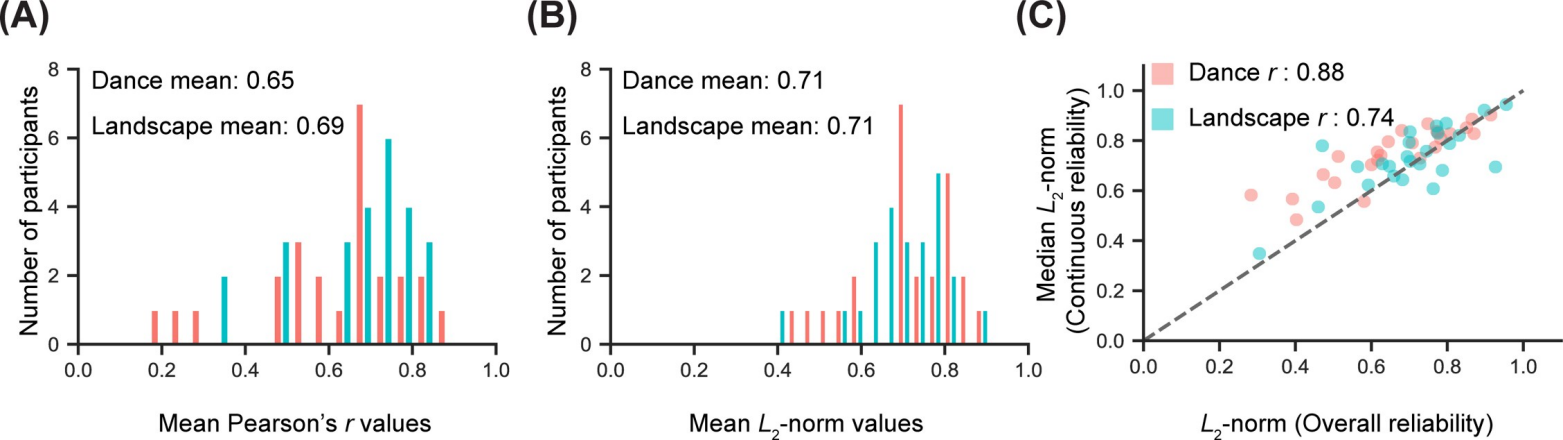
Test-retest reliability of continuous ratings. The distributions of the reliability values both with (A) Pearson correlations and (B) *L*_2-_norm values indicate that most participants showed good test-retest reliability with no difference across categories. (C) There was positive correlation between *L*_2-_norm values for overall ratings and median *L*_2-_norm values for continuous ratings per participants.

Reliability of continuous ratings was in general high but with large variance across participants. To assess the internal reliability of participants’ aesthetic judgments, reliability measures were computed between ratings on first (test) and second (retest) exposures. For each of the 750 test and retest time series pairs (25 observers, 30 movies) a Pearson *r*-score was computed as an estimate of reliability across sessions (Pearson’s *r* has been used frequently to compare time series data [27,48,49]. An average reliability score was then calculated for each participant for both categories (Fig 4A; calculated by transforming the *r*-scores into z-scores using Fischer’s r-to-z transform [43,44], averaging, then transforming back to an *r*-score). Across all participants in the *rate* group, average reliability for Dance videos was *r* = 0.65 (95% CI 0.58–0.71), and for Landscape *r* = 0.69 (95% CI 0.64–0.74).

On average, mean *r-*scores for dance and landscape clips were quite similar, with most individual participants being similarly reliable for the two categories. A Wilcoxon Signed-Ranks showed that there was no difference across categories in continuous rating reliability as calculated by mean *r-*score values per participant (Z = 107, *p*>0.05).

As Pearson correlations might result in inflated reliability estimates due to the presence of autocorrelations in time series data, we also calculated an *L*_2_-norm (Euclidian distance, Eq 1) dissimilarity measure between test and retest time series scaled to vary between -1 (maximum possible difference) and 1 (identical).

$$L_{2}-norm=\sqrt{\sum {\left(X_{i}-Y_{i}\right)}^{2}}$$ (1)

Average *L*_2_-norm reliability scores were computed for each participant in the *rate* group (Fig 4B); the mean and confidence intervals of these scores across participants were Dance Mean = 0.71 (95% CI 0.66–0.76), Landscape Mean = 0.71 (95% CI 0.67–0.75)]. A Wilcoxon Signed-Ranks test showed that participants’ *L*_2-_norm reliability did not differ across categories (Z = 162, *p*>0.05). The distributions of the r-scores and the *L*_2-_norm values for each participant are depicted in S1 Fig.

Furthermore, we also computed reliability for overall ratings by treating each subject’s test and retest overall rating values as vectors and computing the distance between these two vectors using the *L*_2_-norm measure [50]. The average reliability score for *rate* and *view* groups’ overall ratings were as follows Rate|Dance Mean = 0.66 (95% CI 0.60–0.72), Rate|Landscape Mean = 0.70 (95% CI 0.65–0.76), View|Dance Mean = 0.72 (95% CI 0.66–0.77), View|Landscape Mean = 0.73 (95% CI 0.69–0.77. A comparison of reliability for overall ratings (*L*_2-_norm scores) and continuous ratings (median *L*_2-_norm scores) revealed strong positive relationships for both stimulus categories (dance: *r* = 0.88, *p*<0.001, landscape: *r* = 0.74, *p*<0.001; Fig 4C). Thus, observers who were less reliable in their continuous rating traces were also less reliable in their overall judgments.

### Quantifying temporal variability

The degree of dynamic change observed in individual continuous traces was quantified by computing a root-mean-squared derivative of each continuous rating trace (rmsd; Eq 2).

$$rmsd=\sqrt{\frac{1}{n}\overset{n}{\underset{i=1}{\sum }}{\left(\nabla t\right)}^{2}}$$ (2)

**Fig 5:**
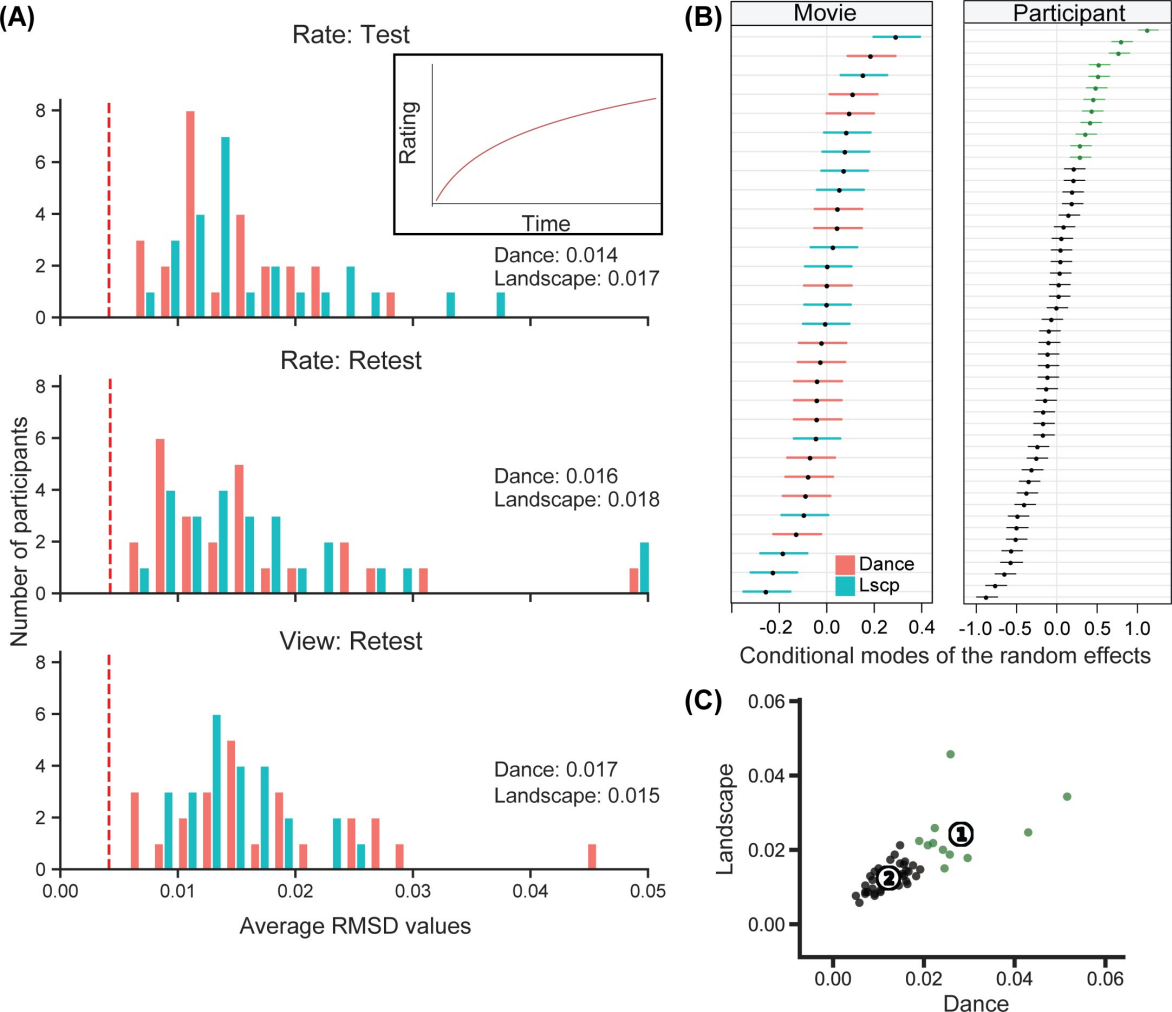
Temporal variability in the continuous ratings. (A) Distributions of mean rmsd values for participants are quite similar across sessions and categories and generally higher than the rmsd value obtained from monotonic increase (inline panel) over the entire clip (red dashed lines). (B) 95% prediction intervals for the random effect estimates of 30 movie clips and 50 participants show that the amount of temporal variability differed more across participants than movies. (Note different x-axes scales for panels.) (C) K-means clustering analysis with median rmsd values resulted in 2 different clusters of participants (shown with green and black). Note that the participants in the first cluster (green-fast responders) have the highest random effects conditional modes.

Overall, the distributions of mean rmsd values across participants were quite similar across sessions and categories (Fig 5A). To aid interpretation, an rmsd score was computed for a simulated logarithmic monotonic increase over the entire clip (Fig 5A, inline panel and vertical dashed red lines). This score was below even the lowest average rmsd value. Thus, even the participants with the least temporal variation produced more dynamic change (on average) than a simple logarithmic increase over the course of the movie. Using LMM regression with the retest session rmsd scores from each group as the dependent variable, we calculated the degree to which rmsd temporal variation was affected by stimulus category or group. This LMM resulted in a significant category by group interaction (Table 2) (B = -0.05, *SE* = 0.02, *t* = -2.77, p = 0.008)

**Table 2:** Results of the linear mixed-model for log transformed rmsd scores as well as a Tukey corrected break down of significant interaction.

**Fixed effects**	***Estimate***	***SE***	***CI***	***t***	***p***
(Intercept)	-4.26	0.06	-4.39 –-4.14	-66.19	**<0.001**
Category (Dance vs Landscape)	0.00	0.03	-0.06–0.05	-0.09	0.926
Group (Rate vs View)	-0.02	0.06	-0.14–0.10	-0.31	0.758
Category x Group	-0.05	0.02	-0.09 –-0.01	-2.77	**0.008**
**Random Effects**					
Residual variance (σ^2^)	0.12	By participant variance in category (τ11)			0.01
Random intercept variance by participant	0.18	Random slope and intercept correlation (ρ01)			0.38
Random intercept variance by item	0.02				
Marginal R^2^ / Conditional R^2^ *	0.009 / 0.640				
	**Tukey Contrasts of LMM Interaction**				
	***Estimate***	***SE***	***t***	***p***	
Rate|Dance—View|Dance	-0.14	0.14	-1.00	0.748	
Rate|Dance—Rate|Landscape	-0.11	0.07	-1.53	0.427	
Rate|Dance—View|Landscape	-0.04	0.13	-0.32	0.989	
View|Dance—Rate|Landscape	0.03	0.13	0.24	0.995	
View|Dance—View|Landscape	0.09	0.07	1.37	0.523	
Rate|Landscape—View|Landscape	0.06	0.11	0.55	0.946	

* Marginal: Variance explained by the fixed factors, Conditional: Variance explained by the fixed and random factors

On the other hand, differences in rmsd scores attributable to participants or movies revealed marked differences in temporal variability across participants, with less discernable structure related to individual movie clips (Fig 5B). 95% prediction intervals for the LMM random effect estimates of different stimuli included zero for 22 of 30 movies (~70%); 95% prediction intervals for random effect estimates of different participants included zero for only 15 of 50 participants (~30%). Thus, participants‘ individual characteristics had more influence on the amount of dynamic change than did stimuli characteristics.

A clustering analysis on rmsd scores identified two groups of participants with different response styles. K-means clustering [51], implemented in Scikit learn with a squared-Euclidean-distance measure [52] was applied to the set of all 50 participants’ median rmsd scores from both the dance and landscape categories. A 2-cluster solution was the best fit to the data (Silhouette score = 0.582). The first cluster included 12 participants identified as showing higher temporal variation; the second cluster contained 38 participants that had lower temporal variation in their continuous ratings (Fig 5C). The 12 participants in Cluster 1 corresponded to the participants showing the highest rmsd random effect estimates in the LMM analysis (green highlight in Fig 5B). Note that for the *rate* group, only data from the retest session was used, allowing all 50 participants to be grouped together; a separate cluster analysis on both test and retest data for the *rate* group only showed very similar clustering patterns (2 clusters; 6 and 19 people in each cluster respectively).

### Measuring “shared taste” for both overall judgments and continuous ratings

**Fig 6:**
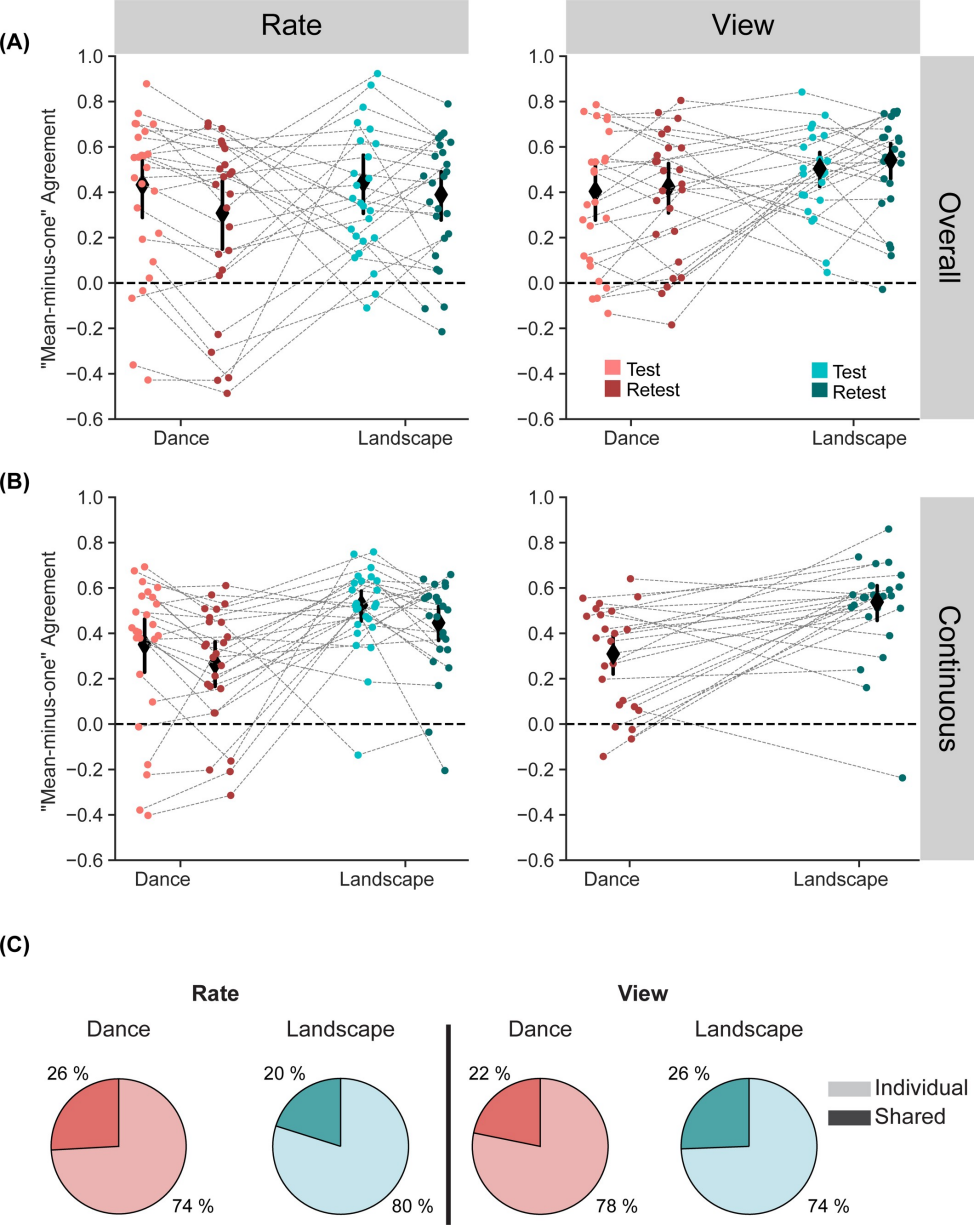
Agreement across participants with “mean-minus-one” (MM1) and variance decomposition. (A) Overall rating agreement with MM1: *Rate* group’s agreement decreased in the retest session whereas *view* group did not show this session effect. (B) Continuous rating agreement with MM1: Agreement is higher for landscape videos for both groups and retest session agreement is lower for the *rate* group. (Error bars show 95% confidence intervals calculated with the z-transformed r values, horizontal dashed line indicated zero) (C) The proportion of repeatable variance that is attributable to individual taste is higher than the proportion of variance that is attributable to shared taste for both categories and groups.

In addition to characterizing participants’ response variability over time, we also characterized variability *across* participants, and found evidence for strong individual differences (Fig 6). A “mean-minus-1” (MM1) measure of agreement (see *Methods, Analysis*) was used to quantify the degree to which different participants had similar aesthetic reactions to the movies (e.g. “shared taste”), for both the overall (Fig 6A) and continuous (Fig 6B) ratings. For overall ratings by the *rate* group, average MM1 values were Test: Dance MM1 = 0.43 (95% CI 0.29–0.56), Retest|Dance MM1 = 0.31 (95% CI 0.15–0.45), Test: Landscape MM1 = 0.44 (95% CI 0.31–0.56) and Retest|Landscape MM1 = 0.39 (95% CI 0.28–0.49). For overall ratings of the *view* group, average MM1 values were Test|Dance MM1 = 0.40 (95% CI 0.28–0.52), Retest|Dance MM1 = 0.42 (95% CI 0.31–0.53), Test|Landscape MM1 = 0.50 (95% CI 0.43–0.58) and Retest|Landscape MM1 = 0.54 (95% CI 0.46–0.62). Particularly for the dance stimuli, there were even individual observers with negative MM1 scores, indicating that their overall ratings were negatively correlated with group average evaluations. For comparison, aesthetic judgments of static images produced MM1 scores of 0.85 for faces, 0.60 for natural landscapes, 0.38–0.40 for architecture, and 0.31 for artworks [17]. Thus aesthetic judgments for the video stimuli used in this experiment contained a level of shared taste lower than what was observed for photographs of natural kinds, more similar to the level observed for artifacts of human culture (with the potential exception of the *view* groups’ landscape judgments). Partitioning of variance of participant responses (e.g.[17,45]) into shared versus individual components (Fig 6C) revealed a similar pattern: both dance and landscape stimuli led to levels of shared taste that was less than photographs of landscapes, but more than photographs of architecture.

Agreement for overall ratings changed from test to retest sessions, but in a manner dependent on whether observers made continuous ratings in the test session or not. An LMM regression analysis of MM1 agreement scores for the overall ratings (see Methods, *Analysis*) found no main effects of stimulus category, session or group, but did reveal a session by group interaction effect (B = 0.04, *SE* = 0.01, *t* = 2.85, *p* = 0.006). (S1 Table). Tukey corrected post-hoc comparisons revealed a decrease in retest session agreement values for only the *rate* group (Test|Rate vs Retest|Rate, *t* = 2.96, *p* = 0.02).

We also carried out a partitioning of the repeatable variance of the overall data [45]. The proportion of repeatable variance in the overall ratings attributable to shared taste was much lower than the proportion of the individual taste and was very similar across groups and categories (Fig 6C).

Across-observer agreement scores for continuous ratings revealed a similar degree of individual variability (Fig 6B), but were affected both by stimulus category and by session. For the *rate* group, average MM1 values were Test|Dance MM1 = 0.35 (95% CI 0.23–0.46), Retest|Dance MM1 = 0.27 (95% CI 0.17–0.36), Test|Landscape MM1 = 0.52 (95% CI 0.45–0.59) and Retest|Landscape MM1 = 0.45 (95% CI 0.28–0.49). For the *view* group, average MM1 values were Retest|Dance MM1 = 0.31 (95% CI 0.22–0.39) and Retest|Landscape MM1 = 0.54 (95% CI 0.46–0.61). An LMM regression analysis of the MM1 scores for the *rate* group’s continuous ratings revealed a main effect of session (B = 0.05, *SE* = 0.02, *t* = 2.78, *p* = 0.006) and category (B = -0.12, *SE* = 0.06, *t* = -2.03, *p* = 0.05) (S2 Table), indicating that agreement was lower in the retest session (compared to the test session) and higher for landscape videos (compared to dance videos).

### Characterizing individual differences with respect to mood, traits and category preferences

In order to better understand potential sources of individual variation, overall ratings and degree of temporal variability we have collected a set of measures across individuals. Multiple regression analyses were used to test the degree to which participants’ overall ratings were predictable from mood and trait measures, including positive and negative affect (PANAS), state and trait anxiety (STAI), ability to experience pleasure (SHAPS), and aesthetic responsiveness (AREA). The results of this regression indicated that positive affect scores significantly predicted higher overall ratings (*B* = 0.44, *t* = 3.24, *p*<0.01) and that combined, the mood and trait measures were able to capture 40% of the variance in average overall ratings (Multiple R^2^ = 0.40, Adjusted R^2^ = 0.32, *F*(6, 43) = 4.79, *p* < .01) (S3 Table). Similarly, we tested whether the degree of temporal variability in continuous ratings was related to the mood and personality measures and found that while there was a negative relationship between SHAPS scores and mean rmsd scores (*B* = -0.36, *t* = -2.48, *p* = 0.02), overall predictability of rmsd scores was low (Multiple R^2^ = 0.20, Adjusted R^2^ = 0.09, *F*(6, 43) = 1.82, *p* = 0.11) (S4 Table).

Additionally, overall aesthetic ratings reflected observers’ self-reported category preferences (reported *prior* to exposure to the stimuli). Using regression, we found that participants’ self-reported preference for landscapes in general predicted their mean overall ratings for landscape videos (*F*(1, 48) = 4.72, *p* = 0.03, *R*^2^ = 0.09, *B* = 0.30, *SE* = 0.05, *t* = 2.17), and their self-reported preference for dance in general predicted their mean overall ratings for dance videos (*F*(1, 48) = 5.59, *p* = 0.02, *R*^2^ = 0.10, *B* = 0.32, *SE* = 0.32, *t* = 2.37). We computed similar regressions with the category liking scores and rmsd scores and found that landscape liking scores predict average rmsd variance scores for landscape videos (*F*(1, 48) = 5.48, *p* = 0.02, *R*^2^ = 0.10, *B* = 0.32, *SE* = 0.00, *t* = 2.34). However, we did not find a relationship between self-reported dance preference score and mean rmsd scores for the dance videos (*F*(1, 48) = 0.12, *p*>0.05, *R*^2^ = 0.00, *B* = 0.05, *SE* = 0.00, *t* = 0.34).

## Discussion

Aesthetic experiences evolve dynamically in time, yet are often studied through the lens of single summary judgments with static stimuli. Using two different types of video stimuli (dance performances and artistic landscape videos) and a continuous response paradigm, we found strong individual differences in the nature of temporally evolving aesthetic experiences. Participants, not movie clips, were the primary factor governing the amount of temporal variation in continuous responses, such that a subset of participants could be characterized as “fast responders.” Furthermore, while most participants produced highly reliable overall aesthetic judgments of video clips (test-retest), reliability in moment-to-moment ratings varied markedly across participants, while still being strongly correlated with reliability for overall judgments. Furthermore, unlike studies that have reported strong shared taste across participants for static images of natural landscapes and faces [17,28,53], we found lower levels of shared taste for landscape and dance videos, more similar to that previously reported for cultural artifacts. Finally, across several comparisons (overall ratings, shared taste, temporal variability) we found no compelling evidence that the continuous report of felt enjoyment during stimulus presentation altered participants’ aesthetic experiences.

A primary aim of this study was to characterize the dynamics of continuous ratings of dynamic stimuli. Therefore, instead of averaging rating time series across participants and using averaged time courses for further analyses [29,54], we chose to employ novel methodologies to characterize individual differences. We found that participants expressed different amounts of temporal variation, varying along a continuum from “fast responders” with highly dynamic responses to “slow integrators” signaling changes in their state after integrating over a longer time period.

These participant profiles were consistent across sessions, stimulus categories and even across different clips. In contrast, differences in the amount of temporal variation consistently attributable to individual movies were much less pronounced. A clustering analysis based on participants’ median temporal variation (rmsd) scores supported this classification. Additionally, inspection of average (rmsd) scores suggest that even slow integrators tended to have more variation than a logarithmic increase or decrease over the duration of the video clip. Note that a LMM analysis of rmsd scores found a weak category by group interaction (more variation for *rate* group for landscape and more variation for *view* group for dance clips), there is no principled reason for the observed direction of the interaction. Further work would be needed to assess the reliability existence of such result.

Surprisingly, none of our participant-level measures were related to the degree of dynamic responding; yet this is clearly an important and dominant individual difference that future studies employing continuous ratings must contend with. It should be noted that our data do not speak to whether differences across participants in degree of dynamic responding reflect actual differences in the rate of change of personal aesthetic experience, or participants’ ability or willingness to report faster dynamics.

Most participants produced highly reliable overall aesthetic judgments of video clips across test-retest sessions. This is in agreement with previous reports of highly stable judgments of images for healthy young adults [17,55]. Reliability of continuous ratings varied more markedly across participants, though was fairly consistent across categories. This suggests that the degree of reliability over repeated presentations of videos may be another strongly individual characteristic. The high correlation between overall and continuous reliability measures supports this claim, potentially hinting at similar sources driving these judgments. Note that both methods used to quantify the degree of reliability [Pearson correlations (more sensitive to global changes) and *L*_2_-norm measure (a good indicator of local changes and the magnitude of the continuous ratings)] led to the same general conclusion.

The literature on repeated presentation of single images tends to predict either overall increases or decreases in liking. On the other hand, Park et al [25] found increased preference for faces and decreased preference for landscapes after repeated presentation, suggesting that image category also plays a role. We found no consistent change in overall aesthetic ratings for either of our categories across repeated presentations. This highlights a potential difference between videos and images of landscapes. One possibility is that videos would require more repetitions than images to show a consistent increase or decrease. Future research is needed to investigate whether additional repetitions would lead to increases, decreases, or other consistent changes in preference ratings of video stimuli.

In general, there was low agreement across people for preferences of video clips. However, while there was no difference across categories in the agreement values for overall ratings, the degree of agreement for continuous ratings for landscape videos was higher than for dance videos. Previous studies found lower across observer agreement for images of artworks and architecture relative to landscapes and faces [17,28]. Such shared preferences for natural categories (faces and landscapes) are likely a result of information processing that is highly conserved across individuals. Evaluations of cultural artifacts (e.g. artworks, architecture) rely more on individual characteristics and varying sources of information, potentially on account of their reduced behavioral relevance for most individuals [17]. Surprisingly, the degree of shared taste observed for the video stimuli in the current study was lower than that observed previously for natural kinds (with the potential exception of landscapes in the *view* group). One possible explanation for this lower agreement is that participants engaged with both video categories as ‘artistic’ stimuli, despite the fact that they contained landscapes or bodies, both natural categories. Across-observer agreement for continuous ratings, while lower than still images, was affected by stimulus category; people were more diverse in their moment-to-moment evaluations of dance clips as compared to landscape clips. This is somewhat surprising, given that the dance clips contained more time-locked events and narrative structure that could have been a basis for changes in continuous evaluations. On the contrary it appears that shared interpretations, such as increased liking when a new perspective on a landscape came into view, influenced moment-to-moment ratings of landscape videos more than for dance. Note that the lack of a significant category difference in agreement for overall ratings is perhaps not surprising as the clips were selected from a larger pool to be roughly matched in their variance in a separate group of participants. Yet, this makes the differences in continuous agreement results more notable. We also observed reduced agreement in the retest session for the *rate* group. It is possible that by asking participants to make both continuous and overall judgments twice, their additional reflection on the material led to more individual assessments in their second judgments of the video clips. However, such a conclusion may be premature without additional study.

By collecting both continuous ratings and summary judgments we aimed to explore the relationship between them. Can a summary judgment capture a fundamentally dynamic experience or are these two measures tapping into different processes? The results of the agreement analysis provide some evidence that a post-stimulus summary judgment can diverge from the moment-by-moment experience. We found that for dance, continuous agreement was lower than overall agreement whereas for landscapes continuous agreement was higher than overall agreement: although people agreed more in the moment-to-moment assessment of landscape than of dance, but this did not lead to higher overall agreement. Overall aesthetic assessments are thus not solely determined by the moment-to-moment liking. If they were, then we would expect much higher agreement for overall assessments of landscapes, or at least a consistent pattern across the two categories.

Across several different measures, we found no consistent evidence that making continuous ratings had an effect on the nature of observers’ aesthetic experiences, as proxied by the overall ratings. This suggests that with minimal practice, people are capable of dealing with the attentional demands of behaviorally reporting aesthetic judgements continuously during stimulus presentation. This observation is also supported by a previous study that collected ratings of continuous emotional responses to amusing and sad films and found that making continuous ratings did not disrupt behavioral or neural emotional measures [26]. One potential caveat comes from the mixed results of the agreement (MM1) analysis, where agreement changed from test to retest session in a manner dependent on the presence of the continuous rating task. Thus, some caution in interpretation is warranted.

With respect to average preferences, landscape videos were liked more than dance videos. It is well documented that aesthetic preferences for nature scenes tend to be higher compared to urban scenes [56] or other categories (faces, architecture, artworks) [17]. On the other hand, studies using dance as stimuli reported that the level of expertise influences affective responses to dance [57,58]. The fact that our participants had no formal expertise in dance may explain why they tended to prefer landscape videos. Note that differences in average motion energy cannot explain this category differences, as the distribution of motion energy was similar for the two categories.

One promising future direction for understanding the nature of continuous rating dynamics would be to identify events in the videos and investigate how changes in ratings relate to perceived event structure. In the current study, we did not perform such an analysis due to the absence of a clear narrative structure in our clips. Future studies with different types of stimuli, or with individualized annotations of event structure, may be useful for understanding the sources of the observed individual differences.

## Conclusions

Aesthetic experiences, like many other psychological phenomena, are temporally unfolding. Here, we show that continuous responses paired with dynamic stimuli can produce a more nuanced understanding of aesthetically pleasing experiences, and reveal the idiosyncratic nature of individuals’ ongoing experiences of the same visual stimulus. By comparing responses across observers who continuously rated videos with observers who only viewed videos, this work also clarifies an outstanding methodological issue with the use of continuous responses. The techniques developed here can be used to investigate aesthetic experiences with a wide variety of stimuli, and can also be applied in the study of emotion and decision making. Moreover, the collection of continuous responses along with neuroimaging or physiological methods is a critical step toward understanding the neural processes supporting such temporally evolving behaviors.

## Supporting information

S1 Fig Reliability between test and retest continuous rating time series. The boxplots show the distributions of **A)** Pearson’s r and **B)** *L*_2_-norm scores for each participant for dance and landscape categories. The degree of reliability was different from person to person. However, most participants showed similar levels of reliability across different categories.
(TIF)

S1 Movie Representative example video clip for dance category. (MP4)

S2 Movie Representative example video clip for landscape category. (MP4)

S1 Table Results of the linear mixed-model for MM1 scores calculated with overall ratings as well as a Tukey corrected break down of significant interaction for Session by Group. (DOCX)

S2 Table Results of the linear mixed-model for MM1 scores calculated with continuous ratings. (DOCX)

S3 Table Results of the multiple regression with mean overall ratings and questionnaire scores. (DOCX)

S4 Table Results of the multiple regression with mean rmsd ratings and questionnaire scores. (DOCX)
