# Supplementary figures and images for: Continuous ratings of movie watching reveal idiosyncratic dynamics of aesthetic enjoyment
Source: PLoS One. 2019 Oct 25;14(10):e0223896. doi: 10.1371/journal.pone.0223896 (PMC6814238; doi:10.1371/journal.pone.0223896)

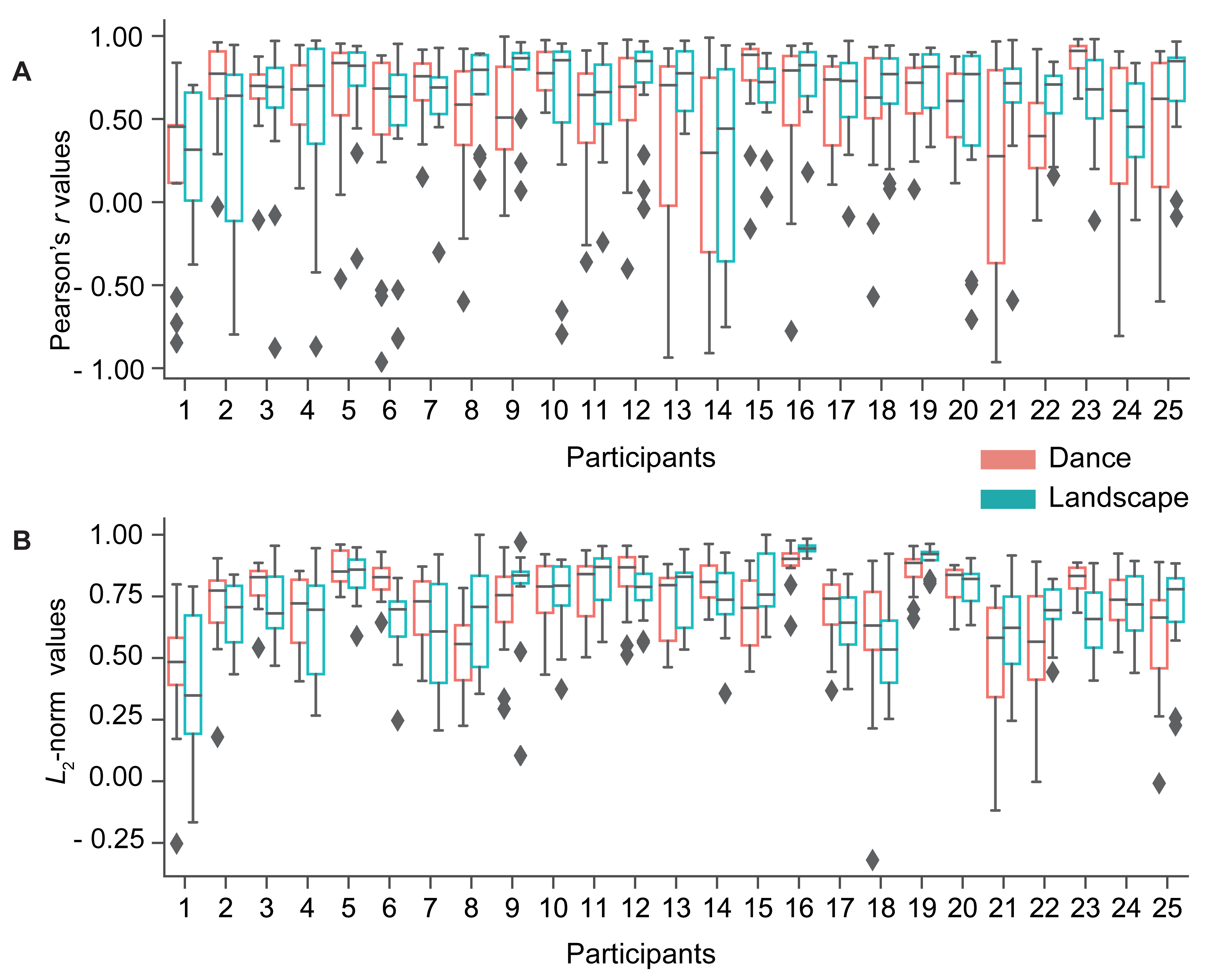

Supplement: S1 Fig — The boxplots show the distributions of A) Pearson’s r and B) L2-norm scores for each participant for dance and landscape categories. The degree of reliability was different from person to person. However, most participants showed similar levels of reliability across different categories. (TIF) [file pone.0223896.s001.tif]
