# Supplementary material for: Continuous ratings of movie watching reveal idiosyncratic dynamics of aesthetic enjoyment
Source: PLoS One. 2019 Oct 25;14(10):e0223896. doi: 10.1371/journal.pone.0223896 (PMC6814238; doi:10.1371/journal.pone.0223896)
Supplement: S1 Table — (DOCX) [file pone.0223896.s004.docx]

| **S1 Table**: Results of the linear mixed-model for MM1 scores calculated with overall ratings as well as a Tukey corrected break down of significant interaction for Session by Group | | | | | |
| --- | --- | --- | --- | --- | --- |
|  | **Mean minus 1 scores for overall ratings (z transfomed)** | | | | |
| **Fixed effects** | ***Estimate*** | ***SE*** | ***CI*** | ***t*** | ***p*** |
| (Intercept) | 0.46 | 0.04 | 0.39 – 0.54 | 11.72 | **<0.001** |
| Session (Test vs Retest) | 0.02 | 0.01 | -0.01 – 0.04 | 1.34 | 0.187 |
| Category (Dance vs Landscape) | -0.05 | 0.03 | -0.11 – 0.01 | -1.69 | 0.098 |
| Group (Rate vs View) | -0.05 | 0.04 | -0.12 – 0.03 | -1.17 | 0.247 |
| Session x Category | 0.01 | 0.01 | -0.01 – 0.03 | 1.20 | 0.238 |
| Session x Group | 0.04 | 0.01 | 0.01 – 0.06 | 2.85 | **0.006** |
| Category x Group | 0.02 | 0.03 | -0.03 – 0.08 | 0.76 | 0.454 |
| Session x Category x Group | 0.01 | 0.01 | -0.02 – 0.03 | 0.56 | 0.578 |
| **Random Effects** |  |  |  |  |  |
| Residual variance (σ^2^) | 0.02 | By participant variance in session (τ11) | | | 0 |
| Random intercept variance by participant | 0.07 | Random slope and intercept correlation (category) (ρ01) | | | 0.3 |
| Random intercept variance by item | 0.04 | Random slope and intercept correlation (session)(ρ01) | | | 0.38 |
| Marginal R^2^ / Conditional R^2^ * | 0.048 / 0.826 | | | | |
| * Marginal: Variance explained by the fixed factors, Conditional: Variance explained by the fixed and random factors | | | | | |
|  | **Tukey Contrasts of LMM Interaction for Session x Group Interaction** | | | | |
|  | ***Estimate*** | ***SE*** | ***t*** | ***p*** |  |
| Rate\|Test x Rate\|Retest | 0.11 | 0.04 | 2.96 | **0.024** |  |
| Rate\|Test x View\|Test | -0.02 | 0.09 | -0.24 | 0.995 |  |
| Rate\|Test x View\|Retest | -0.06 | 0.08 | -0.71 | 0.893 |  |
| Rate\|Retest x View\|Test | -0.13 | 0.08 | -1.53 | 0.430 |  |
| Rate\|Retest x View\|Retest | -0.17 | 0.08 | -2.10 | 0.169 |  |
| View\|Test x View\|Retest | -0.04 | 0.04 | -1.07 | 0.708 |  |
