# Supplementary material for: Continuous ratings of movie watching reveal idiosyncratic dynamics of aesthetic enjoyment
Source: PLoS One. 2019 Oct 25;14(10):e0223896. doi: 10.1371/journal.pone.0223896 (PMC6814238; doi:10.1371/journal.pone.0223896)
Supplement: S2 Table — (DOCX) [file pone.0223896.s005.docx]

| **S2 Table**: **Results of the linear mixed-model for MM1 scores calculated with continuous ratings** | | | | |  |
| --- | --- | --- | --- | --- | --- |
|  | **Mean Minus 1 scores for continuous ratings (z transfomed)** | | | | |
| **Fixed effects** | ***Estimate*** | ***SE*** | ***CI*** | ***t*** | ***p*** |
| (Intercept) | 0.47 | 0.06 | 0.35 – 0.59 | 7.53 | **<0.001** |
| Session (Test vs Retest) | 0.05 | 0.02 | 0.01 – 0.08 | 2.78 | **0.006** |
| Category (Dance vs Landscape) | -0.12 | 0.06 | -0.24 – -0.00 | -2.03 | **0.05** |
| Group (Rate vs View) | -0.04 | 0.03 | -0.10 – 0.01 | -1.46 | 0.151 |
| Session x Category | 0.00 | 0.02 | -0.04 – 0.03 | -0.16 | 0.874 |
| Category x Group | 0.02 | 0.03 | -0.03 – 0.07 | 0.71 | 0.479 |
| **Random Effects** | | | | | |
| Residual variance (σ^2^) | 0.46 | By participant variance in category (τ11) | |  | 0.02 |
| Random intercept variance by participant | 0.03 | Random slope and intercept correlation (ρ01) | |  | 0.24 |
| Random intercept variance by item | 0.09 |  | |  |  |
| Marginal R^2^ / Conditional R^2^ * | 0.026 / 0.252 | | | | |
| * Marginal: Variance explained by the fixed factors, Conditional: Variance explained by the fixed and random factors | | | | | |
