# Supplementary material for: Continuous ratings of movie watching reveal idiosyncratic dynamics of aesthetic enjoyment
Source: PLoS One. 2019 Oct 25;14(10):e0223896. doi: 10.1371/journal.pone.0223896 (PMC6814238; doi:10.1371/journal.pone.0223896)
Supplement: S3 Table — (DOCX) [file pone.0223896.s006.docx]

| **S3 Table. Results of the multiple regression with mean overall ratings and questionnaire scores** | | | | | | | | | | |
| --- | --- | --- | --- | --- | --- | --- | --- | --- | --- | --- |
|  | **B** | **SE B** | **CI B** | | | **β** | **t** | **p** |  |  |
| (Intercept) | -0.50 | 0.24 | -0.98 | - | -0.02 | 0.00 | -2.08 | **0.04** |  |  |
| PANAS (positive) | 0.02 | 0.01 | 0.01 | - | 0.03 | 0.44 | 3.24 | **0.002** |  |  |
| PANAS (negative) | 0.00 | 0.01 | -0.01 | - | 0.01 | 0.01 | 0.08 | 0.93 |  |  |
| SHAPS | 0.02 | 0.01 | 0.00 | - | 0.05 | 0.24 | 1.92 | 0.06 |  |  |
| STAI (State | -0.01 | 0.00 | -0.01 | - | 0.00 | -0.34 | -1.92 | 0.06 |  |  |
| STAI (Trait) | 0.00 | 0.00 | 0.00 | - | 0.01 | 0.27 | 1.76 | 0.09 |  |  |
| AREA | 0.00 | 0.00 | 0.00 | - | 0.00 | 0.19 | 1.48 | 0.15 |  |  |
| Multiple R^2^ | 0.40 |  |  |  | *F*(6, 43) | |  | 4.79 |  |  |
| Adjusted R^2^ | 0.32 |  |  |  | *p* |  |  | 0.00 |  |  |
|  |  |  |  |  |  |  |  |  |  |  |
