# Supplementary material for: Continuous ratings of movie watching reveal idiosyncratic dynamics of aesthetic enjoyment
Source: PLoS One. 2019 Oct 25;14(10):e0223896. doi: 10.1371/journal.pone.0223896 (PMC6814238; doi:10.1371/journal.pone.0223896)
Supplement: S4 Table — (DOCX) [file pone.0223896.s007.docx]

|  |  |  |  |  |  |  |  |  |  |  |
| --- | --- | --- | --- | --- | --- | --- | --- | --- | --- | --- |
| **S4 Table: Results of the multiple regression with mean rmsd ratings and questionnaire scores** | | | | | | | | | | |
|  | **B** | **SE B** | **CI B** | | | **β** | **t** | **p** |  |  |
| (Intercept) | 0.04 | 0.02 | 0.00 | - | 0.07 | 0.00 | 2.29 | **0.03** |  |  |
| PANAS (positive) | 0.00 | 0.00 | 0.00 | - | 0.00 | 0.15 | 0.93 | 0.36 |  |  |
| PANAS (negative) | 0.00 | 0.00 | 0.00 | - | 0.00 | -0.22 | -1.37 | 0.18 |  |  |
| SHAPS | 0.00 | 0.00 | 0.00 | - | 0.00 | -0.36 | -2.48 | **0.02** |  |  |
| STAI (State | 0.00 | 0.00 | 0.00 | - | 0.00 | 0.02 | 0.09 | 0.93 |  |  |
| STAI (Trait) | 0.00 | 0.00 | 0.00 | - | 0.00 | -0.08 | -0.45 | 0.65 |  |  |
| AREA | 0.00 | 0.00 | 0.00 | - | 0.00 | 0.27 | 1.83 | 0.07 |  |  |
| Multiple R^2^ | 0.20 |  |  |  | *F*(6, 43) | |  | 1.82 |  |  |
| Adjusted R^2^ | 0.09 |  |  |  | *p* |  |  | 0.12 |  |  |
